# Selective Attention and Information Processing Speed in Graves’ Disease: Stroop Interference Effect

**DOI:** 10.7759/cureus.14072

**Published:** 2021-03-23

**Authors:** İskender Ekinci, Esra Ekinci, Mitat Buyukkaba, Ahmet Cinar, İrem Kirac Utku, Hanise Ozkan, Muhammed Tunc, Abdulbaki Kumbasar, Omur Tabak

**Affiliations:** 1 Internal Medicine, Health Sciences University, Kanuni Sultan Suleyman Training and Research Hospital, Istanbul, TUR; 2 Social Services Department, Directorate of Woman and Family Services, Istanbul Metropolitan Municipality, Istanbul, TUR; 3 Internal Medicine, Arnavutkoy State Hospital, Istanbul, TUR; 4 Internal Medicine, Kanuni Sultan Suleyman Training and Research Hospital, Istanbul, TUR; 5 Internal Medicine, Bezmialem Vakif University, Istanbul, TUR

**Keywords:** graves' disease, stroop interference effect, selective attention, information processing speed

## Abstract

Background

Cognitive functions are affected by thyroid hormones. In this study, we aimed to investigate the selective attention and information processing speed in thyrotoxic Graves’ disease.

Methodology

This study was conducted among 40 patients with thyrotoxic Graves’ disease and age and gender-matched 40 healthy controls. Stroop Color and Word test were applied to healthy controls once and to patients with Graves’ disease during thyrotoxic and euthyroid periods. Stroop interference effect was calculated.

Results

The mean age was 34.67 ± 11 in the Graves’ group and 34.72 ± 9.16 in the control group (p > 0.05). The number of errors and self-corrections in Stroop Color and Word test was higher in patients with thyrotoxic Graves’ disease than both patients with euthyroid Graves’ disease and healthy controls (p < 0.05). Stroop interference effect was significantly longer in patients with thyrotoxic Graves’ disease than both patients with euthyroid Graves’ disease and healthy controls (p < 0.05). All parameters obtained from the Stroop Color and Word test including errors, self-corrections, and Stroop interference effect were similar in patients with euthyroid Graves’ disease and healthy controls.

Conclusions

Selective attention was impaired and information processing speed was slow in patients with thyrotoxic Graves’ disease, and these findings were associated with age and educational level. After becoming euthyroid through antithyroid medication, these pathological findings returned to normal levels. Additionally, Stroop interference effect was significantly decreased when patients with Graves’ disease became euthyroid.

## Introduction

Graves’ disease, the most common cause of hyperthyroidism, is an autoimmune disease associated with elevated circulating thyroid hormone levels [1]. Hyperactivity, palpitations, agitation, asthenia, heat intolerance, hand tremors, hyperreflexia, increased bowel movements, weight loss despite increased appetite, muscle weakness, irregular menstrual cycles, and mental problems can be observed due to elevated circulating thyroid hormone levels in patients with this condition [2].

Thyroid hormones play an important role in brain function. It has been reported that elevated thyroid hormone levels in hyperthyroidism caused oxidative stress, thereby leading to neuron damage, and patients with hyperthyroidism had decreased gray matter volume, wherein the cerebral metabolism and neurotransmitter system were affected by thyroid hormone levels [3]. Changes in the levels of neurotransmitters such as dopamine, which is associated with motivation; noradrenaline, which is associated with attention, concentration, and wakefulness; and serotonin, which is associated with mood, sleep, nutrition, and aggression may affect several neuropsychiatric functions, especially cognitive functions, thus leading to cognitive-behavioral pathologies in individuals with thyroid disease [4]. Therefore, these patients may easily get angry and exhibit mental pathologies such as irritability, anxiety, impulsiveness, emotional instability, inattention, inability to concentrate, sleeplessness, restlessness, and distractibility [5].

The Stroop Color and Word test, which is closely associated with frontal lobe functions, is a neuropsychological test that measures selective attention and information processing speed and analyzes cognitive processes [6]. In other words, this test evaluates the ability to inhibit cognitive interference that occurs when the processing of a particular stimulus feature prevents the simultaneous processing of a second stimulus feature. This test is based on the difference between the ink color in which a word is printed and the color expressed by the word. The ability to name the color despite the tendency to read is related to the ability and flexibility to shift attention and behavior [7]. Compared to the case when the ink color in which a word is printed and the color referred by the word are the same, if the said ink color is different from the color that the word refers to, the tendency to focus on naming the color and reading the name of the color causes a delay, which constitutes the Stroop interference effect [8].

This study aimed to (1) determine the selective attention level and information processing speed with the Stroop Color and Word test in patients recently diagnosed with thyrotoxic Graves’ disease who had not yet started treatment, (2) observe the changes in attention levels and information processing speed when these patients became euthyroid under treatment, and (3) examine the relationship of selective attention level and information processing speed with thyroid hormone and autoantibody levels, as well as the psychopathological processes such as anxiety and depression.

## Materials and methods

Study design

This study was approved by the Ethics Committee of Health Sciences University, Kanuni Sultan Suleyman Training and Research Hospital (No: KAEK/2018.5.15). This prospective case-control study was conducted at the Internal Medicine Department of Health Sciences University, Kanuni Sultan Suleyman Training and Research Hospital. A total of 40 patients with Graves’ disease and 40 age and gender-matched healthy controls were enrolled between June 1, 2018 and April 1, 2019.

Patient characteristics and data collection

The patient group comprised those who had thyrotoxicosis and were diagnosed with Graves’ disease at the time of admission to the outpatient clinic, and the control group comprised healthy volunteers without any disease. Patients aged between 18 and 55 years, who were recently diagnosed with Graves’ disease and were not yet started on antithyroid drug therapy, who had at least eight years of education, and who were color-aware and not colorblind were enrolled in the patient group. Patients who were illiterate, colorblind, or those who did not know colors; those aged <18 or >55 years; those with comorbidities other than Graves’ disease; those with a known history of psychopathological or neurological disorders or those receiving treatment for such disorders; those with alcohol dependence; and those with a history of head trauma and any pathologies related to vision were excluded from the study.

Demographic data (age, gender, marital status, education level, and smoking status) of the subjects were recorded. The patient group was started on antithyroid drug therapy (methimazole) and followed up at the outpatient clinic at one-month intervals. Free T3 (fT3), free T4 (fT4), thyroid stimulating hormone (TSH), antithyroglobulin (antiTG), antithyroid peroxidase (antiTPO), and TSH receptor antibody (TRAb) levels were noted at the first admission visit when the patients had thyrotoxicosis and at the first visit when they became euthyroid.

Stroop Color and Word test

This test was performed in the patient group both at the visit when the patients had thyrotoxicosis and at the first visit when euthyroidism was observed, whereas the control group was subjected to the Stroop Color and Word test only at the visit conducted for inclusion in the study. The test comprised four white cards, each 14 × 21.5 cm in size. Each card comprised six lines and each line contained four items. The test was applied in the following five stages:

Task one: Reading the names of colors printed in black ink on white background on the first card.

Task two: Reading the words, wherein the ink color is different from the color referred by the word.

Task three: Naming the colors of the circles printed in color.

Task four: Naming the colors of some words that are printed in color but do not refer to a color.

Task five: Naming the colors of words, wherein the ink color is different from the color referred by the word.

The time taken to complete each stage was measured by a stopwatch. The said time was evaluated as the time between the start of saying the first word or color and saying the last word or color on the card. The total time taken to complete all stages was obtained by adding up the times taken to complete each stage.

The number of errors and corrections made was noted for each stage. The total number of errors and corrections was calculated.

The first stage determines the level of reading speed, whereas the third and fourth stages show the speed of naming the color. The Stroop interference effect was calculated as the difference between the times taken to complete stages 5 and 1.

Beck Anxiety Inventory: This scale is a Likert-type, self-reported scale with 21 items scored between 0 and 3 [9]. The score range is 0-63, and a high total score indicates higher severity of anxiety experienced by an individual.

Beck Depression Inventory: This scale comprised 21 items, 15 related to psychology and six related to somatic symptoms, with each item scored between 0 and 3 [10]. The highest score is 63, and a higher score indicates a higher level and severity of depression.

Statistical analysis

Statistical analysis was performed using SPSS for Windows 26.0 (IBM Corporation, Chicago, IL, USA). Categorical variables were expressed with numbers and percentages and numerical variables with mean ± standard deviation values. Shapiro-Wilk test was used to determine how a variable is distributed. Chi-square test was used to compare categorical data between the groups. In comparing the two groups in terms of numerical variables, Student’s t-test was used when the variables showed normal distribution, and Mann-Whitney U test when they showed non-normal distribution. Paired samples t-test or Wilcoxon test was used in the analysis of dependent quantitative data. Pearson’s correlation analysis was performed for variables with normal distribution and Spearman correlation analysis for variables with non-normal distribution.

## Results

Overall, 40 patients with Graves’ disease and 40 healthy controls were included in the study. Both groups were similar in terms of mean age, gender distribution, marital status, smoking status, and education levels (Table 1). The scores obtained from Beck Anxiety Scale and Beck Depression Scale were higher in patients with thyrotoxic Graves’ disease than in the control group, wherein the former also exhibited a higher incidence of anxiety and depression.

**Table 1:** Demographic data of the groups.

		Patient group	Control group	P-Value
Age, years		34.67 ± 11	34.72 ± 9.16	0.983
Gender	Male, n	16	19	0.499
	Female, n	24	21	
Marital status	Married, n	31	26	0.217
	Single, n	9	14	
Smoker, n		18	15	0.496
Education time, year		11.7 ± 4.4	12.4 ± 5.2	0.492
Beck Anxiety Scale score		20 ± 12.89	10.8 ± 6.44	<0.001
Presence of anxiety, n (%)		22 (55)	9 (22.5)	0.003
Beck Depression Scale score		17.55 ± 10.05	10.95 ± 6.33	0.001
Presence of depression, n (%)		17 (42.5)	7 (17.5)	0.013

The laboratory results at the first visit when patients with Graves’ disease had thyrotoxicosis and at the visit when patients first became euthyroid are presented in Table 2. Free T3, fT4, antiTPO, antiTG, and TRAb levels significantly decreased, whereas TSH levels significantly increased in patients who became euthyroid under treatment.

**Table 2:** Laboratory findings in Graves’ disease. fT3: free T3; fT4: free T4; TSH: thyroid stimulating hormone; AntiTPO: antithyroid peroxidase; AntiTG: antithyroglobulin; TRAb: TSH receptor antibody

	Patient group, thyrotoxic status	Patient group, euthyroid status	P-Value
fT3, pg/mL	11.43 ± 7.02	3.35 ± 0.93	<0.001
fT4, pg/mL	3.37 ± 1.85	1.12 ± 0.34	<0.001
TSH, uIU/mL	0.01 ± 0.02	0.93 ± 1.44	<0.001
AntiTPO, IU/mL	249.71 ± 204.49	156.56 ± 153.25	<0.001
AntiTG, IU/mL	615.16 ± 1081.02	315.55 ± 614.92	0.007
TRAb, IU/mL	9.14 ± 11.18	3.36 ± 3.26	<0.001

Results of the Stroop Color and Word test performed during both the thyrotoxic and euthyroid periods in patients with Graves’ disease and results of the Stroop Color and Word test of the control group are presented in Table 3. The completion times of the entire test as well as tasks 1, 2, and 5 had shortened; the number of self-corrections made in tasks 3, 4, and 5 and in the entire test had decreased; and the number of errors made in task 5 and the total number of errors made throughout the entire test had decreased when patients with thyrotoxic Graves’ disease became euthyroid. The time taken to complete four cards except the second card and the time taken to complete the entire test were longer and the number of corrections made on the fifth card as well as the total number of corrections made during the entire test were higher in patients with thyrotoxic Graves’ disease than in the control group. Comparing the patients with Graves’ disease who were euthyroid with the control group, all results were similar except the time taken to complete task 1.

**Table 3:** Comparison of the Stroop Color and Word Test results in thyrotoxic and euthyroid status of the patients with Graves’ disease and control group. p1: Wilcoxon test, p-value between “patient group, thyrotoxic status” and “patient group, euthyroid status”; p2: Mann-Whitney U test, p-value between “patient group, thyrotoxic status” and “control group”; p3: Mann-Whitney U test, p-value between “patient group, euthyroid status” and “control group”

		Patient group, thyrotoxic status	Patient group, euthyroid status	p1	Control group	p2	p3
1.task	Errors	0	0	-	0	-	-
	Self-corrections	0	0	-	0	-	-
	Time, s	11.58 ± 4.44	10.68 ± 3.3	0.003	8.99 ± 1.5	0.004	0.023
2.task	Errors	0	0	-	0.02 ± 0.15	-	-
	Self-corrections	0.15 ± 0.42	0.07 ± 0.34	0.317	0.1 ± 0.3	0.699	0.423
	Time, s	11.89 ± 4.2	12.49 ± 5.23	0.572	10.77 ± 2.87	0.544	0.358
3.task	Errors	0	0	-	0.02 ± 0.15	-	-
	Self-corrections	0.17 ± 0.44	0.02 ± 0.15	0.034	0.12 ± 0.33	0.717	0.092
	Time, s	11.24 ± 3.4	10.72 ± 3.27	0.054	9.63 ± 1.74	0.01	0.242
4.task	Errors	0.1 ± 0.63	0.02 ± 0.15	0.655	0.05 ± 0.22	0.579	0.559
	Self-corrections	0.7 ± 1.09	0.12 ± 0.33	0.001	0.3 ± 0.46	0.122	0.057
	Time, s	20.37 ± 7.48	16.28 ± 5.06	<0.001	15.23 ± 4.17	<0.001	0.436
5.task	Errors	0.65 ± 1.29	0.07 ± 0.26	0.002	0.2 ± 0.4	0.418	0.107
	Self-corrections	1.7 ± 1.6	0.8 ± 0.96	0.001	0.62 ± 0.97	0.001	0.260
	Time, s	27.73 ± 8.92	22.35 ± 5.68	<0.001	20.91 ± 5.31	<0.001	0.319
All tasks	Errors	0.75 ± 1.46	0.1 ± 0.3	0.004	0.32 ± 0.65	0.620	0.07
	Self-corrections	2.72 ± 2.26	1.02 ± 1.16	<0.001	1.1 ± 1.39	0.001	0.915
	Time, s	82.84 ± 24.51	72.53 ± 18.76	<0.001	65.55 ± 13.69	0.001	0.199
Stroop interference effect, s		16.14 ± 7.29	11.67 ± 4.89	<0.001	11.91 ± 5.31	0.011	0.885

Stroop interference effect significantly decreased when patients with Graves’ disease became euthyroid. Stroop interference effect was significantly higher in patients with thyrotoxic Graves’ disease than in the control group; however, there was no difference between the control group and euthyroid patients with Graves’ disease in terms of Stroop interference. Gender (male: 14.54 ± 4.65, female: 17.21 ± 8.55; p: 0.557), smoking status (smoker: 18.28 ± 7.93, nonsmoker: 14.39 ± 6.37; p: 0.140), marital status (married: 16.43 ± 8.04, single: 15.16 ± 3.89; p: 0.899), presence of anxiety (patients with anxiety: 17.6 ± 7.46, those without anxiety: 14.36 ± 6.85), and presence of depression (patients with depression: 15.52 ± 6.11, those without depression: 16.6 ± 8.16; p: 0.957) did not show a significant difference in terms of Stroop interference effect in patients with thyrotoxic Graves’ disease.

In the correlation analysis performed in patients with thyrotoxic Graves’ disease, age and length of education had a significant correlation with Stroop interference effect and the time taken to complete the Stroop Color and Word test, the results of which are presented in Table 4. No significant correlation was observed in correlation analysis performed with the scores obtained from Beck Anxiety Scale, Beck Depression Scale, and all laboratory parameters (fT3, fT4, TSH, antiTPO, antiTG, and TRAb) in terms of the time taken to complete the Stroop Color and Word test and Stroop interference effect (p > 0.05, for all).

**Table 4:** Correlation analysis between Stroop Color and Word Test findings and age and education time in thyrotoxic Graves’ disease. Spearman correlation analysis was performed

		Age, year	Education time, year
1.task time, s	r	0.409	-0.755
	p	0.009	<0.001
2.task time, s	r	0.345	-0.747
	p	0.029	<0.001
3.task time, s	r	0.140	-0.764
	p	0.390	<0.001
4.task time, s	r	0.209	-0.888
	p	0.195	<0.001
5.task time, s	r	0.109	-0.881
	p	0.503	<0.001
All tasks time, s	r	0.269	-0.975
	p	0.093	<0.001
Stroop interference, s	r	-0.175	-0.541
	p	0.279	<0.001

## Discussion

In this study, selective attention was impaired and information processing speed was slowed in patients with thyrotoxic Graves’ disease, but these pathological findings returned to normal levels when euthyroidism was achieved with treatment. The results of the Stroop Color and Word test were not associated with anxiety and depression in patients with Graves’ disease who exhibited these psychopathologies more frequently than the healthy population. However, the results of this test were closely associated with age, especially with education level. In addition, thyroid hormone and autoantibody levels, gender, smoking, and marital status did not have a relationship with the results of the Stroop Color and Word test.

Patients with hyperthyroidism tended to have problems in decision-making under ambiguous conditions, and this pathology could stem from dopamine dysfunction and metabolic disorders in the frontal cortex and limbic system [4]. Changes in dopamine metabolism are thought to have negative effects on attention and concentration. In a study conducted with 27 patients with Graves’ disease, attention and concentration tests were worse in the patient group than in the group with normal thyroid functions [11]. In the same study, it was also reported that elevated thyroid hormone levels could directly cause damage in attention pathways via an unknown mechanism, or the accelerated metabolism could lead to increased synthesis and secretion of various hormones that have an effect on attention and cause deterioration in attention and concentration in these patients. According to a study that examined various patient groups with thyroid dysfunction in terms of wakefulness, orientation, and executive control, negative outcomes were observed in terms of wakefulness in patients with hypothyroidism and in terms of wakefulness and executive control in patients with hyperthyroidism, whereas there were no negative outcomes in subclinical hypo and hyperthyroidism groups [12]. In the present study, parameters that assessed attention in the Stroop Color and Word test were worse in patients with Graves’ disease, which is consistent with the literature.

The onset of dementia, a syndrome characterized by deterioration in memory and cognitive functions, was observed earlier in patients with hyperthyroidism, and suppressed TSH and elevated fT4 levels were also effective in the development of this syndrome [13]. In another study, overt hyperthyroidism, especially elevated fT4 levels, led to an increased risk of dementia [14]. In a study conducted with a geriatric patient group with low TSH levels and without dementia, low TSH levels were associated with poor cognitive functions and administration of methimazole slowed down cognitive function impairment [15]. Similarly, in the study by Jensovsky et al., normalization of the low TSH led to improved cognitive functions as well as improved verbal, visual, and general memory [16]. In the Stroop Color and Word test, which was used as a measure of cognitive functions in the present study, the aforementioned test results were worse (impaired) in the group of patients with Graves’ disease than in the healthy population, whereas the aid impaired functions returned to normal levels when patients became euthyroid after antithyroid drug therapy.

In the study by Alvares et al., the tests related to attention and concentration were not associated with fT4 levels and free thyroxine index in patients with Graves’ disease [11]. According to the study by Yuan et al., there was a negative correlation between executive control and fT4 in patients with hypothyroidism and a positive correlation between the same in patients with hyperthyroidism [12]. In our study, the parameters that measured attention and information processing speed did not have any relationship with thyroid hormone and autoantibody levels.

In the present study, anxiety and depression were more frequent in the group of patients with Graves’ disease than in the healthy population, which is consistent with the literature [5,12]. More than half of our patient group had anxiety (55%) and nearly half of the same group had depression (42.5%). However, neither the presence of anxiety and depression nor the scores obtained from the tests that were performed for both psychopathologies showed any correlation with the results of the Stroop Color and Word test. There are several studies stating that these psychopathologies may have an effect on the Stroop effect; however, there are also studies reporting otherwise [12,17].

Aging leads to some changes in brain structure and function, and these changes affect higher cognitive functions such as language processing and decision-making, especially the main cognitive functions such as attention and memory [18]. The time taken to perform any given task (such as the Stroop Color and Word test) in the presence of a distracting stimulus is longer in older adults than in young individuals. It is believed that the main reason for this prolonged response in older adults is a slowdown in information processing speed rather than decreased selective attention [18]. In the present study, there was a close relationship between age and the first two tasks in Stroop Color and Word test, whereas there was no relationship between Stroop interference effect and age.

Education is robustly associated with the level of cognitive function [19]. Early education, social learning experiences, and the skills necessary to pursue intellectual challenges throughout life contribute toward the development of cognitive functions [20]. In this study, there was a close relationship between education level and the parameters associated with selective attention and information processing speed. Moreover, response time in the test applied had shortened with a higher education level, and there was a significant correlation between the length of education and Stroop interference effect.

The first limitation of this study was the relatively small sample size. Another limitation was that the geriatric population was not included. Impairment in cognitive functions is an expected process in the geriatric patient group, and the extent to which these functions will be affected when Graves’ disease is present may be a new study topic for researchers working in this field. Similarly, studies in which neurotransmitter levels affecting cognitive functions are measured and changes in brain tissue caused by elevated thyroid hormone levels are observed with cranial imaging may help clarify this issue.

## Conclusions

In conclusion, this study showed that selective attention was impaired and information processing speed slowed down in patients with Graves’ disease, but this negative effect disappeared when euthyroidism was achieved with treatment. The extent to which a person is affected by Graves’ disease was closely associated with education level, but not with thyroid hormone or antibody levels. The number of relevant studies is very limited in the literature, and we think that our study has contributed toward the elucidation of this issue.
